# Effect of Occupational Health and Safety Training for Chinese Construction Workers Based on the CHAID Decision Tree

**DOI:** 10.3389/fpubh.2021.623441

**Published:** 2021-05-21

**Authors:** Zhonghong Cao, Tao Chen, Yuqing Cao

**Affiliations:** ^1^School of Accounting, Wuhan Qingchuan University, Wuhan, China; ^2^School of Management, Wuhan University of Science and Technology, Wuhan, China; ^3^School of Physics, Huazhong University of Science and Technology, Wuhan, China

**Keywords:** construction workers, occupational health and safety, training effect, CHAID, five-factor method

## Abstract

**Background:** Occupational health and safety (OHS) training is an important way to prevent construction safety risks. However, the effectiveness of OHS training in China is questionable. In this study, the CHAID (chi-squared automatic interaction detection) decision tree, chi-square analysis, and correlation analysis were used to explore the main, secondary, weak, unrelated, and expectation factors affecting the effectiveness of training. It is the first to put forward the “five-factor method” of training effectiveness. It is found that training effectiveness is positively correlated with job responsibilities, OHS training, and job satisfaction. It is also significantly related to job certificate, training time, training method, and working time. However, the effectiveness of training has nothing to do with personal age, marital status, educational level, job type, and whether or not they have experienced industrial accidents. And the workers on site expect the enterprise to provide security and opportunities such as physical safety, training and learning, and future career development. The results show that OHS system training should be strengthened in the construction industry, and classified training should be carried out according to post responsibility, training methods, job satisfaction, and working hours.

## Introduction

According to statistics, in China, there are about 3,000 construction workers injured and killed every year (1). In recent decades, the awareness of occupational safety and health risks in the construction industry has been increasing. However, despite the substantial improvement, the accident rate is still significantly higher than that in most other industries (2). With the entry of construction enterprises into the international competitive market, the health and safety risks of field workers have also increased dramatically (3). Therefore, the risks of construction occupation, especially the OHS injury risks of construction site workers, have attracted more and more attention from construction enterprise managers (4, 5). According to the research literature on the application of the OHS management system in the construction industry, the main measures of OHS risk management are to strengthen the study and training of work posts, pay attention to the research of operation process technology, and prevent the risk influence factors (6). It can be seen that OHS training is the research focus of safety training in the construction industry. However, at present, there are few studies on the training effect of the OHS management system for construction workers, and less research on the actual situation of the construction industry (7). Therefore, it is necessary to analyze the effectiveness and influencing factors of the OHS training system in the construction industry and explore the strategies and methods to improve the effectiveness of OHS training in the construction industry.The effectiveness of training refers to the benefits obtained by the company and employees from the training. For employees, the benefits mean learning new knowledge or skills. For companies, the benefits include an increase in sales and customer satisfaction. The effectiveness of training is often reflected in the training results. In recent years, the management of OHS training in construction enterprises has become one of the core issues concerned by various countries. On the restrictive factors of implementing safety measures in construction contractors, it is found that the main factors affecting the safety practice of contractors are the perfection of occupational safety and health management, and lack of training knowledge in safety management, etc. (8). Therefore, it is urgent to professionalize the construction sector and implement a “risk prevention culture” among the personnel participating in construction activities (9). Therefore, this paper puts forward the hypothesis H1: the effect was positively correlated with the role. The research on the intervention effect of risk awareness training in the construction industry shows that the knowledge and attitudes of construction workers after a one-hour safety course can be improved within 3 months (10). In particular, the post guidance training of apprentices has significantly improved the effect of on-site management (11). At the same time, the owner-manager is the key to ensuring the safe working conditions of apprentices. The test results show that it is feasible to carry out standardized training for the owner's management personnel and training company to prevent apprentice injury (12).

Many factors are affecting the effectiveness of OHS training. Research on suicide prevention of construction workers shows that mental health is the fundamental factor of OHS (13–15). Gender issues and organizational structures in the workplace may affect OHS practices. However, the construction industry is a high-risk occupation with a large number of male employees, and the risk of major injury and death of male employees is high (16, 17). There is a great correlation between the aging of construction site workers and physical strength and occupational safety and health, and there is a negative correlation between aging and occupational safety and health (OSH). It is necessary to promote differentiated management of occupational safety and health for elderly employees (18, 19). Therefore, the authors try to propose a new approach to improve the effectiveness of training by combining factors of age, gender, and job category to explore more aspects of safety risk prevention and risk awareness intervention training. Therefore, this paper puts forward the hypothesis H2: the effect was positively correlated with training. H3: the effect was positively correlated with satisfaction.

Moreover, the physical health factors of construction workers are closely related to OHS (20). Effective prevention measures such as infectious diseases in the workplace need to consider occupational risk factors and control occupational exposure, to protect the health of workers (21). In the prevalence rate and related factors of occupational injury among construction workers, gender, education level, safety training, personal protective equipment, and other factors were a significantly static correlation (22). The unsafe behavior of construction workers is the main factor causing accident injury (23), and the effective intervention of the OHS management system (OHS-MS) can improve active safety behavior (24). Accordingly, through strengthening the OHS training, it has become the focus of risk management of construction enterprises to improve the effect of OHS training for construction workers to reduce the number of deaths in construction accidents and ensure the OHS of construction workers (25). The cross-sectional survey of construction workers found that the injuries with high incidence were mostly cuts and night-shift injuries. Therefore, strengthening education, publicity, and training, guaranteeing their rights to safety and protection at work, as well as legislative implementation, can help to reduce the occurrence of occupational injuries (26). The grass-roots construction workers need reasonable and effective training methods. Therefore, it is necessary to explore the main factors affecting safety and improve the training effect in combination with the actual situation of the construction industry.

Others had found that working environment factors have a greater impact on outdoor workers and their safety (27), and there is a positive correlation between night fatigue work and mortality of construction workers (28). Moreover, extreme climate, sunlight, and ultraviolet radiation also have significant effects on workers' health (29–32). At the same time, safety climate factors (33, 34) and construction noise also have effects on workers' health (35). Therefore, one of the main purposes of this paper is to study the working time and working environment of construction workers.

Besides, the OHS of construction projects require corresponding cost investment (36), and the health and safety performance of enterprise projects is related to a variety of influencing factors (37). Safety management ability is an important quality of construction personnel. Although the acquisition ability of safety knowledge of construction personnel is not significantly related to age and experience, there are significant differences in safety management ability in professional classification (38). The latest research found that new construction equipment (such as GPS and physiological sensors) can improve the management level of occupational safety and health (39). Through the use of modern support technology, lean construction, safe construction, and management can be achieved (40–42). The world has paid great attention to OHS in the era of industry 4.0, which will bring about a new paradigm shift, which will have a profound impact on OHS management (43). Therefore, it is also a concern of the authors to study whether or not it is also a more effective way to improve OHS with the attention of construction workers to new technology, new materials, new technology, new equipment, and other information, as well as the timeliness of training.

Based on the current situation of OHS training in the construction industry, this study carried out corresponding investigation and research to find out the factors affecting the effectiveness of OHS training and then explored ways and methods to improve the effectiveness of OHS training. Based on understanding the current situation of OHS in the global construction industry (44), taking the effect of OHS training on construction sites as the object, the qualitative analysis results were obtained by using the methods of a questionnaire survey and decision tree intelligent discriminant analysis, to provide countermeasures for the application of OHS management systems in OHS training of the construction industry (2).

The decision tree intelligent discriminant analysis method is a kind of classification regression tree algorithm (45), which is widely used in medical diagnosis (46), biological analysis (47), deep learning (48) and optical application analysis (49), and also has many applications in data comparative analysis (50) and classification importance analysis (51). In the field of engineering, the decision tree is mainly used in equipment improvement (52), geotechnical engineering special new analysis (53), building damage assessment (54), and engineering modeling (55). Therefore, this paper chooses the CHAID decision tree method for analysis.

To sum up, through the research of relevant literature, it is found that there are many kinds of literature about safety training, but there is little special research literature and examples on the safety training of construction workers. It is rarer to combine chi-square analysis, related factor analysis, and decision tree analysis into empirical research and form a comprehensive analysis. There are many studies on safety behavior factors in the existing literature, and most of them are from the perspective of managers. There are few empirical methods to conduct one-to-one research at the grass-roots level. This paper adopts a one-to-one questionnaire, which has high reliability and effectiveness. In particular, this paper is based on the CHAID discriminant analysis modeling analysis, based on previous studies to fill a certain blank area. To find out the various factors that affect the effectiveness of OHS training in construction sites, and the importance of these factors, this paper makes some useful exploration.

## Methods

### Population

In this study, a variety of statistical analysis methods and questionnaires were used as data collection tools. A total of 22 questions related to OHS training (including 21 multiple-choice questions and one open-ended suggestion question) were designed in this questionnaire. Twenty five topics were initially designed in the tutor team's academic meeting. After training and consulting tutors and technical experts, 22 questionnaire questions were determined. Then, 10 construction workers were preliminarily investigated, and the answer options were modified. Finally, the formal questionnaire was formed by Volume. The questionnaire was designed and surveyed from December 2018 to July 2020. To ensure the representativeness of the questionnaire survey, we selected four representative regions (provinces) in China, namely East China (Shandong Province), South China (Hainan Province), central China (Hubei Province), and North China (Hebei Province), and interviewed 393 workers face-to-face at random, forming 374 valid questionnaires. The number of samples is more than 15 times the number of questions in the questionnaire, which is in line with the basic quantity of the questionnaire.

### Statistical Analysis

To ensure the reliability and effectiveness of the data set, see Appendix Tables 1, 2. The source and analysis of the data are as follows: Firstly, it is widely distributed in the four representative regions in China, and the proportion of samples in each region (P1~P4) is roughly balanced (19.3~38.2%). Secondly, the proportion of each sample of 16 items (E1~E16) selected from the four regions was balanced (3.2~9.4%). Finally, 393 workers were interviewed face-to-face on the construction site with corresponding anonymous and confidential measures.

According to the plan made by the statistical level of the number of workers, the obtained data are evaluated by a descriptive method, and the data are determined by statistical analysis, and the effective questionnaire is modeled and analyzed. The variables used in this study are described in Table 1. The Codes and Variables in Table 1 are from Q1~Q22 in Supplementary Material (Investigation on occupational health and safety (OHS) of construction site workers), and the Name is from the dataset of IBM SPSS statistics 23. This variable table design ensures the consistency of variable naming between the questionnaire and the data set, making it more convenient to view.

**Table 1:** Name of variables.

**Codes**	**Name**	**Variables**
Q1	Gender	Your gender
Q2	Age	Your age
Q3	Education	Your level of education
Q4	Marital	Your marital status
Q5	Job	Your job type
Q6	Certificate	Do you have a vocational skill certificate
Q7	Cumulative	Your cumulative working life in the construction industry
Q8	Working days	About the number of working days per week
Q9	Hours	How many hours do you work per day
Q10	Skills	How do you acquire your job skills and knowledge
Q11	Satisfaction	Your job satisfaction
Q12	Industries	Have you worked in other industries
Q13	Information	Do you often pay attention to information
Q14	Accident	Have you ever experienced an accident
Q15	Witnessed	Have you ever witnessed an accident
Q16	Training	Have you received occupational health and safety (OHS) training
Q17	Related training	Have you received other related training
Q18	Recently	When have you recently received occupational
Q19	Effect	What do you think is the effect of recent occupational training
Q20	Type	How do you think OHS training or other related training is more appropriate
Q21	Role	Occupational health and safety training or other related training plays a role in job responsibility
Q22	Suggestions	Do you have any other suggestions

### Procedure

**Figure 1:**
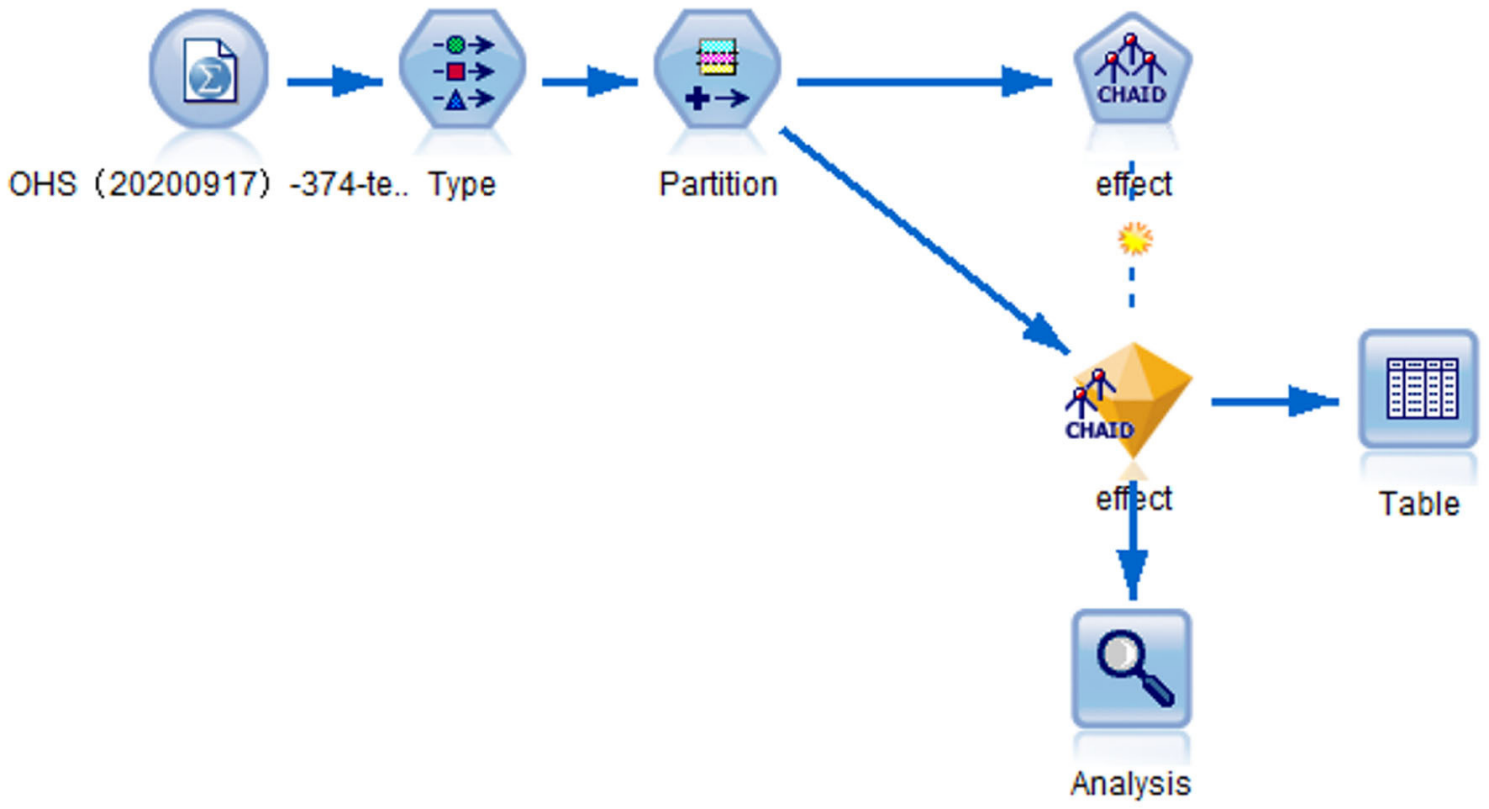
Research methodological model.

The method model used in this study is shown in Figure 1. IBM SPSS statistics 23 and IBM SPSS modeler 18.0 are used for modeling and analysis, and the CHAID decision tree is selected. The effect is the output variables of the model and the rest of the variables are input ones (see Table 1).

## Results

### Analysis of Related Factors

The correlation coefficient is the quantity to measure the linear correlation between variables, which is used to indicate whether there is a correlation between two variables, and how close the correlation is. Typical correlation coefficients are the Pearson correlation coefficient and Spearman correlation coefficient. Generally, the correlation coefficient above 0.7 indicates that the relationship is very close; 0.4~0.7 indicates that the relationship is close; 0.2~0.4 indicates that the relationship is general (33, 40, 56). Correlation analysis has used to find out the related variables with a correlation coefficient >0.2, and describe the correlation between variables. Referring to variable Table 1, the following assumptions are put forward.

H1: The effect was positively correlated with the role.
H2: The effect was positively correlated with training.
H3: The effect was positively correlated with satisfaction.

According to the correlation analysis table (Table 2), the Pearson correlation of effect and role is 0.347, *P*-value = 0.000, indicating that there is a positive correlation between effect and role, so hypothesis H1 holds; Pearson correlation of effect and training is 0.307, *P*-value = 0.000, indicating that there is a positive correlation between effect and training, so hypothesis H2 holds; Pearson correlation of effect and satisfaction = 0.251, *P*-value = 0.000, indicating that there is a positive correlation between effect and satisfaction, so H3 is assumed. Besides, the independent variables with significant but weak relationship strength with the dependent variable effect are certificate, working days, information, related training, recently, and type.

**Table 2:** Correlations.

**Name**	**Effect**		
	**Pearson correlation**	**Sig. (2-tailed)**	***N***
Certificate	0.154^**^	0.003	374
Working days	−0.121^*^	0.019	
Satisfaction	0.251^**^	0.000	
Information	0.146^**^	0.005	
Training	0.307^**^	0.000	
Related training	0.190^**^	0.000	
Recently	0.148^**^	0.004	
Type	0.141^**^	0.006	
Role	0.347^**^	0.000	

** *Correlation is significant at the 0.01 level (2-tailed)*.

* *Correlation is significant at the 0.05 level (2-tailed)*.

It is found that the independent variables with significant but weak relationship strength are certificate, working days, information, related training, recency, and type. The correlation coefficient between these variables and the dependent variable effect is <0.2, so it can be considered that the correlation between these variables and the variable effect is weak or uncorrelated, but because of the value of *P* < 0.05, there exists a significant relationship, so it is only presented in a correlation matrix.

### Intelligent Discriminant Analysis of Decision Tree

The decision tree intelligent discriminant analysis method (45) is widely used in medical diagnosis, biological analysis, deep learning, and optical application analysis, and also has many applications in data comparative analysis and classification importance analysis. In the field of engineering, the decision tree is mainly used in equipment improvement, geotechnical engineering analysis, building damage assessment, and engineering modeling. The output field of the CHAID decision tree is especially suitable for classification variables. Its advantage is that it can generate a multi-branch decision tree, and the target variable can be fixed distance or class. The branch variable and partition value can be determined from the perspective of statistical significance, and then the branching process of the tree is optimized. In the discussion of causality, many levels of input variables can be divided according to the target variables. Therefore, this model is selected as the main analysis method of this empirical data (56).

**Figure 2:**
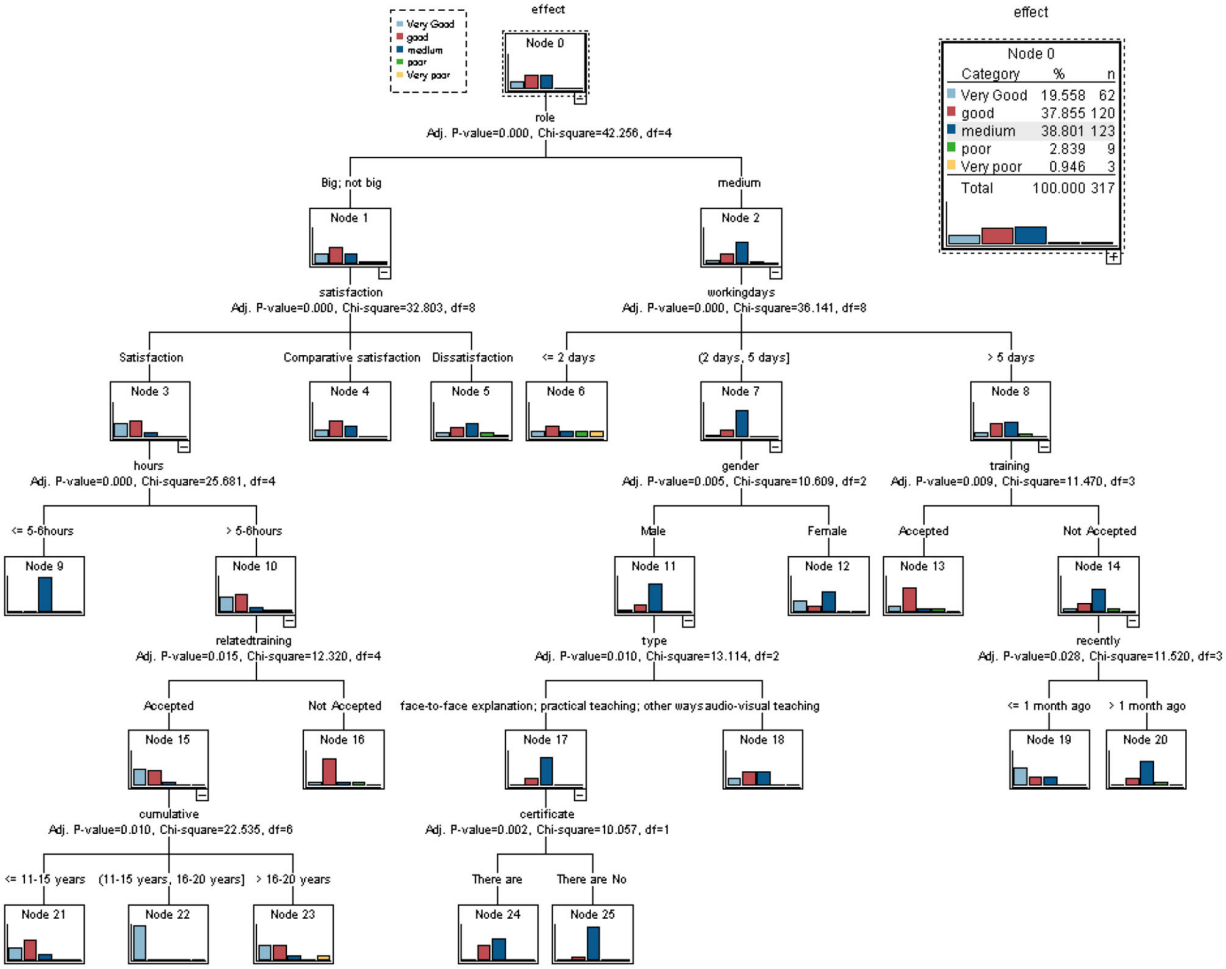
Decision tree (CHAID).

In this paper, all the options of the questionnaire are analyzed by using the decision tree model of SPSS. It is found that some variables can be constructed into corresponding decision tree models (see Figure 2). The decision tree model is divided into five levels. The top variable is the effect, and the first level variable is the role. The second level variables generated in turn are satisfaction and working days. The third level variables are hours, gender, and training. The fourth level variables are related to training, type, and recency. The fifth level variables are cumulative and certificate. All the above variables are closely related to the variable effect. Among the sub-variables of Node 0, the proportion of medium is 38.801%, the proportion of good is 37.855%, and the proportion of Very good is 19.558%. It can be seen that the sum of the proportion of good and Very good is 57.415%, which means that the proportion of the dependent variable effect above good is higher.

### Crosstab and Chi-Square Analysis

See Figure 2 and Table 3, according to the chi-square test analysis of the variable effect ^*^ role (X^2^= 42.256, *P*-value = 0.000, df = 4), it can be seen that there is a significant relationship between effect and role. Similarly, there are significant relationships between role and satisfaction, role and working days, satisfaction and hours, working days and gender, working days and training, hours and related training, hours and related training, gender and type, training, and recency, related training and cumulative, type and certificate.

**Table 3:** Chi-square tests.

**Variable**	**X^**2**^**	***P*-value**	**df**
Effect * role	42.256	0.000	4
Role * satisfaction	32.803	0.000	8
Role * working days	36.141	0.000	8
Satisfaction * hours	25.681	0.000	4
Working days * gender	10.609	0.005	2
Working days * training	11.470	0.009	3
Hours * related training	12.320	0.015	4
Gender * type	13.114	0.010	2
Training * recently	11.520	0.028	3
Related training * cumulative	22.535	0.010	6
Type * certificate	10.057	0.002	1

The analysis of Table 3 shows that the table is mainly used to show the significant relationship between the upper and lower levels of Figure 2 for the convenience of viewing and comparing. The connection between hierarchical branches in the CHAID decision tree is the relationship between variables with a significant relationship.

### Comprehensive Analysis

Based on the above analysis, by using decision tree analysis and chi-square analysis, the relationship between an independent variable and the dependent variable effect is classified into four categories. The first types of variables are role, training, and satisfaction. These variables are not only significantly related to the dependent variable effect (see Table 3 and Figure 2), but also positively correlated with the dependent variable effect (see Table 2). The second types of variables are the certificate, working days, related training, hours, recently, type. These independent variables are significantly and closely related to the dependent variable effect (see Table 2 and Figure 2). The third types of variables are independent variables gender, cumulative, and information. The relationship between these three variables and the dependent variable effect is not significant, but there is a weak relationship (see Table 2 and Figure 2). The fourth types of variables are the remaining variables that are independent of the dependent variable effect. These variables are age, education, marital status, job, skills, industries, accident, witnessed, which have little impact on the effectiveness of training. Besides, the independent variable suggestions represent open-ended questions, among which the most frequently answered one is the need for physical examination, followed by the need for training, and processing resources ranked seventh.

According to the above statistics and analysis, the following results can be obtained.

(1) Training effectiveness is positively correlated with job responsibilities, training, and satisfaction.
(2) The effectiveness of training is significantly correlated with the possession of the certificate, related training, recent training time, and appropriate training method; it was also significantly related to working days and hours per day.
(3) Training effectiveness has a weak correlation with gender, cumulative working years, and whether to pay attention to training information, but it is not significant.
(4) The effectiveness of training has nothing to do with personal age, marital status, and education; it also has nothing to do with job type and past industries; it also has nothing to do with whether they have experienced industrial accidents or seen work-related accidents.
(5) In the OHS training management process, workers are more concerned about personal health (physical examination), participation in training, and career planning, while increasing wages and salaries is not the main expectation factor.

## Discussion

Because there are many sources of the effectiveness of OHS training for construction workers, domestic and foreign scholars have studied from the aspects of personal factors, material status factors, management factors, environmental factors, and technical factors. European and American scholars put forward interdisciplinary (occupational health, industrial health, safety management, ergonomics) technical concepts and methods (57), a reasonable training plan is an effective way to improve knowledge and skills (58), especially mental health training can effectively improve the knowledge, confidence, and attitude of trained personnel (59). However, there are few achievements in the in-depth study from the construction site. To find out the specific factors that affect the effectiveness of OHS training, this paper designs 21 investigation factors and development suggestions based on the actual factors. To ensure the authenticity and credibility of the investigation, the authors went directly to 16 construction sites in China and interviewed the workers face to face for nearly 14 months. Three hundred and ninety three questionnaires were obtained, of which 374 were valid. To analyze and model effectively, this paper adopts IBM SPSS statistics 23 and IBM SPSS modeler 18.0 and selects the CHAID decision tree model with a better classification modeling effect. The decision tree clearly describes the various factors affecting the effectiveness of training and lays the foundation for the follow-up analysis and research. Compared with other research results in the literature review, we can see that, first of all, this study has a more in-depth analysis of influencing factors, and there are more types of factors. Secondly, based on others' research, this paper puts forward the “five-factor method.”

The main contributions of this paper are as follows: first, by using the CHAID decision tree, chi-square analysis, and correlation analysis, the main factors, secondary factors, weak factors, unrelated factors, and expectation factors affecting the effectiveness of training are explored, and the “five-factor method” for training effectiveness is proposed for the first time. The second is to propose appropriate ways to improve the effectiveness of training. The construction industry needs to strengthen the OHS system training for grass-roots field workers and should carry out classified training according to post responsibility, training methods, job satisfaction, and working hours. At the same time, the training should be strengthened for all age groups, educational backgrounds, and post personnel. The factors of on-site management that have nothing to do with the effectiveness of training are revealed.

The “five-factor method” in the research results of this paper mainly includes five aspects. One is to find that the effectiveness of training is positively related to job responsibilities, whether to participate in training, and job satisfaction. These three factors are the main factors affecting the effectiveness of training, which can be called class A factors. The second is that the effectiveness of training is significantly related to having a job certificate, participating in relevant training, recent training time, and appropriate training methods; it is also significantly related to working days per week and working hours per day. These six factors are the secondary factors affecting the effectiveness of training, which can be called class B factors. Thirdly, there is a weak correlation between training effectiveness and gender, cumulative working years, and whether to pay attention to training information, but it is not significant. These three factors are the weak factors affecting the effectiveness of training, which can be called class C factors. Fourth, the effectiveness of training has nothing to do with personal age, marital status, and educational level; it also has nothing to do with the type of post and previous employment; it also has nothing to do with whether or not they have experienced or seen industrial accidents. These seven factors have nothing to do with the influence of training effectiveness, which can be called class D factors. Fifthly, in the OHS training management process, workers are more concerned about personal health (physical examination), participation in training, career planning and salary increase, and other expectations. This kind of factor is called the E factor. We refer to the above five factors as the “five factors method” which affects the effectiveness of training.

The practice shows that the classification management of various factors involved in the construction site and the control of the main factors can improve the effectiveness of training, to improve the health and safety of workers. Although this paper reveals many influencing factors of training effectiveness, and also puts forward reasonable ways to improve training effectiveness, due to the limitations of questionnaire setting options, there must be other influencing factors not taken into account. The later research will increase the investigation factors and conduct further analysis. Besides, there are many ways to improve the safety of construction workers, such as safety training, system construction, executive power, and so on. Therefore, management should grasp the main factors and improve the efficiency of safety training. The effectiveness and main influencing factors of OHS training should also be combined with the government policy documents of different countries and regions, as well as taking corresponding measures and implementing dynamic adjustment of training methods in the different development stages of the construction market.

## Conclusion

The main research results of this paper are to use the high credibility of a one-to-one questionnaire survey on the spot and to model and analyze the empirical data, put forward a comprehensive analysis model based on CHAID, and for the first time, put forward the “five-factor method” of training effectiveness, and explore the improvement and realization methods of the effectiveness of OHS training for construction workers. Through the above analysis, the main conclusions are as follows.

(1) The “five-factor method” of training effectiveness is a method of combining theory with practice in the effective management of construction site workers' training.
(2) The decision tree analysis model based on CHAID is effective to analyze the influencing factors of the effectiveness of training on construction sites.
(3) The OHS system training should be strengthened in the construction industry, and classified training should be carried out according to the post responsibilities, training methods, job satisfaction, and working hours. At the same time, the training should be strengthened for all age groups, educational backgrounds, and post personnel.

The research results of this paper can provide corresponding countermeasures for the training of OHS in China's construction industry, and can also be used for reference for training in similar fields in other developing countries. The practical significance of this research is as follows: First of all, training effectiveness is related to or significant with A, B, and C factors. Managers should distinguish the importance of different types of factors to improve training efficiency. But class E expectations should be taken into account as much as possible. Secondly, class, A factor is the main factor, which has three factors: role, training, and satisfaction. Therefore, managers are required to grasp the main factors. And secondary factors should be considered with enough importance, too. Finally, according to the important factors of different levels, strengthening the OHS training of construction enterprise workers is an effective way to improve the level of construction site occupational health management.

## Data Availability Statement

The datasets presented in this study can be found in online repositories. The names of the repository/repositories and accession number(s) can be found in the article/Supplementary Material.

## Author Contributions

TC: conceptualization, methodology, writing of original draft preparation, supervision, and funding acquisition. ZC: formal analysis, data curation, visualization, and project administration. TC and ZC: validation. ZC and YC: software and writing of review and editing. All authors contributed to the article and approved the submitted version.

## Conflict of Interest

The authors declare that the research was conducted in the absence of any commercial or financial relationships that could be construed as a potential conflict of interest.

## Appendix

**Table A1:** Nomenclature and frequency of the categories of province variables.

**Variable/category codes**	**Variables/category of variables**	**Frequency (%)**
***P***	**Province**	**100.0**
P1	Hubei	38.2
P2	Shandong	24.1
P3	Hainan	18.2
P4	Hebei	19.5

**Table A2:** Nomenclature and frequency of the categories of engineering variables.

**Variable/category codes**	**Variables/category of variables**	**Frequency (%)**
***E***	**Engineering**	**100.0**
E1	Hubei Xiaochang Shidai 1	5.3
E2	Digital Astronomical Museum of Wuhan University of Technology	5.3
E3	Zhonghe Jingcheng	5.3
E4	Xingyiyuan 2	5.9
E5	Hongsha Zongnutan G2-1	8.8
E6	Handan Gongyu	9.1
E7	Jingdian Shijicheng 2	7.2
E8	Wuhan Xingfucun H3	4.8
E9	Yangxin People Hospital	4.0
E10	Gedian Dukeng Suosi	8.0
E11	Honglian Bandao 2and4	5.3
E12	Xiaogan Yangping Fangfan Industrial Park	5.3
E13	Jinan west Exhibition Center	9.4
E14	The outpatient building of Dezhou people's hospital	8.8
E15	Haikou Boyi kindergarten project	4.0
E16	Hebei Mingyue new town phase I	3.2
