# Supplementary material for: Effect of Occupational Health and Safety Training for Chinese Construction Workers Based on the CHAID Decision Tree
Source: Front Public Health. 2021 May 21;9:623441. doi: 10.3389/fpubh.2021.623441 (PMC8175887; doi:10.3389/fpubh.2021.623441)
Supplement: Supplementary file 1 [file Data_Sheet_1.DOC]

**Supplementary**

***Investigation on Occupational Health and Safety (OHS) of***

***Construction Site Workers***

Dear Sir/ Madam,

The purpose of this questionnaire is to provide suggestions for the relevant departments to formulate the policy system of on-the-job training for construction workers based on investigating the current situation of OHS training for construction workers. The results of this questionnaire are only used for academic research. Thank you for participating in this questionnaire!

**Q1:** Your gender.

(1) Male,(2)female

**Q2:** Your age.

(1) Under 18 years old, (2)18-29 years old, (3)30-39 years old, (4)40-49 years old, (5)50-59 years old, (6)60 years old and over

**Q3:** Your level of education.

(1)Primary school, (2)junior high school, (3)senior high school, (4)secondary school, (5)junior college, (6)college and above

**Q4:** Your marital status.

(1) Unmarried, (2)Married, (3)Divorced,(4)Widowed

**Q5:** Your job type.

(1)Woodworking, (2)reinforcing steel, (3)concrete, (4)masonry, (5)painter or decorator, (6)electrician, (7)plumber,(8)repairman, (9)tower, crane(hoist), outdoor elevator or signaller,(10) other types of work

**Q6:** Do you have a vocational skill certificate?

(1)There are. (2)There are No.

**Q7:** Your cumulative working life in the construction industry.

(1) less than one year, (2)1-5 years, (3)6-10 years, (4)11-15 years, (5)16-20 years, (6)more than 20 years

**Q8:** About the number of working days per week.

(1) less than 2 days, (2)2 days,(3) 3 days,(4) 4 days, (5)5 days,(6) more than 5 days

**Q9:** How many hours do you work per day?

(1) Less than 5 hours, (2)5-6hours, (3) 6-7hours,(4) 9-10 hours,(5) more than 10 hours

**Q10:** How do you acquire your job skills and knowledge?

(1)Vocational training, (2)apprenticeship, (3)self-study,(4) other ways

**Q11:** Your job satisfaction.

(1)Satisfaction, (2) Comparative satisfaction,(3) Dissatisfaction

**Q12:** Have you worked in other industries before working in the construction industry?

(1)I have worked in other industries. (2)I have not worked in other industries.

**Q13:** Do you often pay attention to information about new technologies, equipment, or materials in the field of construction?

(1) Concern (2) Not Concern

**Q14:** Have you ever experienced an accident or injury at work?

(1)I've experienced it myself;(2) I haven't experienced it myself.

**Q15:** Have you ever witnessed an accident or injury at work?

(1)Seeing,(2) not seeing

**Q16:** Have you received occupational health and safety (OHS) training?

(1) Accepted (2) Not Accepted

**Q17:** Have you received any other training (except OHS) related to construction positions?

(1) Accepted,(2) Not Accepted

**Q18:** When have you recently received occupational health and safety training or other related training?

(1)1 month ago, (2)3 months ago, (3)6 months ago,(4) 1 year ago

**Q19:** What do you think is the effect of recent occupational health and safety training or other related training?

(1)Very Good,(2) good, (3)medium,(4) poor, (5) Very poor.

**Q20:** How do you think OHS training or other related training is more appropriate?

(1) face-to-face explanation; (2) audio-visual teaching; (3) practical teaching; (4) other ways

**Q21:** In your opinion, occupational health and safety training or other related training plays a role in job responsibility awareness.

(1) Big (2) not big (3) medium

**Q22:** Do you have any other suggestions for this survey?

Please recommend the following: .
